# Novel Omega-3 Fatty Acid Epoxygenase Metabolite Reduces Kidney Fibrosis

**DOI:** 10.3390/ijms17050751

**Published:** 2016-05-18

**Authors:** Amit Sharma, Md. Abdul Hye Khan, Scott P. Levick, Kin Sing Stephen Lee, Bruce D. Hammock, John D. Imig

**Affiliations:** 1 Department of Pharmacology & Toxicology, Medical College of Wisconsin, 8701 Watertown Plank Road, Milwaukee, WI 53226, USA; amitsharma@mcw.edu (A.S.); abhkhan@mcw.edu (M.A.H.K.); slevick@mcw.edu (S.P.L.); 2 Cardiovascular Center, Medical College of Wisconsin, Milwaukee, WI 53226, USA; 3 Department of Entomology & Cancer Center, University of California, Davis, CA 95616, USA; skslee@ucdavis.edu (K.S.S.L.); bdhammock@ucdavis.edu (B.D.H.)

**Keywords:** omega-3 fatty acid, fatty acid epoxide, renal fibrosis, epithelial-to-mesenchymal transition

## Abstract

Cytochrome P450 (CYP) monooxygenases epoxidize the omega-3 polyunsaturated fatty acid (PUFA) docosahexaenoic acid into novel epoxydocosapentaenoic acids (EDPs) that have multiple biological actions. The present study determined the ability of the most abundant EDP regioisomer, 19,20-EDP to reduce kidney injury in an experimental unilateral ureteral obstruction (UUO) renal fibrosis mouse model. Mice with UUO developed kidney tubular injury and interstitial fibrosis. UUO mice had elevated kidney hydroxyproline content and five-times greater collagen positive fibrotic area than sham control mice. 19,20-EDP treatment to UUO mice for 10 days reduced renal fibrosis with a 40%–50% reduction in collagen positive area and hydroxyproline content. There was a six-fold increase in kidney α-smooth muscle actin (α-SMA) positive area in UUO mice compared to sham control mice, and 19,20-EDP treatment to UUO mice decreased α-SMA immunopositive area by 60%. UUO mice demonstrated renal epithelial-to-mesenchymal transition (EMT) with reduced expression of the epithelial marker E-cadherin and elevated expression of multiple mesenchymal markers (FSP-1, α-SMA, and desmin). Interestingly, 19,20-EDP treatment reduced renal EMT in UUO by decreasing mesenchymal and increasing epithelial marker expression. Overall, we demonstrate that a novel omega-3 fatty acid metabolite 19,20-EDP, prevents UUO-induced renal fibrosis in mice by reducing renal EMT.

## 1. Introduction

Chronic kidney disease (CKD) is a progressive disease that results in end-stage renal disease, and there is a need for renal replacement therapy or transplantation. Indeed, CKD is rapidly increasing in industrialized countries due to increases in disorders, such as obesity, and CKD is a major economic healthcare burden [1,2,3].

Tubulointerstitial fibrosis is an early event in progressive renal disease that leads to functional abnormalities and eventual decline of renal function irrespective of the contributions of diverse pathophysiological factors [4,5,6]. Proximal tubular epithelial cells play a central role in renal tubulointerstitial fibrosis [7]. Accumulation of extracellular matrix components and loss of tubular architecture are key features of tubulointerstitial fibrosis. Epithelial mesenchymal transition (EMT) occurs at a critical period in the pathogenesis of tubulointerstitial fibrosis and involves renal tubular epithelial cells transforming phenotypically and functionally into myofibroblasts [8]. This transformation is characterized by a decline in E-cadherin expression and an elevation in α-smooth muscle actin (α-SMA) expression. Therefore, EMT in the kidneys offers a significant therapeutic target, and emphasizes the need to develop approaches to inhibit tubular epithelial cells from undergoing EMT to prevent tubulointerstitial fibrosis.

One such novel approach could be the metabolites of cytochrome P450 (CYP450) monooxygenase pathway that represent the third branch of polyunsaturated fatty acid (PUFA) metabolism [9]. CYP450 monooxygenases can epoxidize PUFAs to generate three-membered oxirane known as epoxides [9]. In the case of arachidonic acid, CYP450 monooxygenases convert this omega-6 PUFA into epoxyeicosatrienoic acids (EETs), which act to regulatecardiovascular function and inflammatory processes to protect the kidney in a number of pathologies [10]. In addition to omega-6 PUFA, CYP450 monooxygenases also converts the omega-3 PUFA eicosapentaenoic acid (EPA) and docosahexaenoic acid (DHA) into novel epoxyeicosatetraenoic (EEQs) and epoxydocosapentaenoic (EDPs) acids, respectively [11,12]. Recent studies demonstrated anti-hypertensive and anti-inflammatory actions of 19,20-EDP, and indicated 19,20-EDP as a novel CYP450 metabolite with promising cardiovascular actions [13,14].

In the present study, we examined the kidney protective and anti-fibrotic action of 19,20-EDP in unilateral ureteral obstruction (UUO), a model for renal tubulointerstitial fibrosis. Our results demonstrated marked kidney protection and anti-fibrotic actions of 19,20-EDP in UUO model. We also demonstrated that the anti-fibrotic action of 19,20-EDP is caused by its ability to reduce renal EMT.

## 2. Results

### 2.1. 19,20-EDP Treatment Attenuates UUO Renal Injury

**Figure 1:**
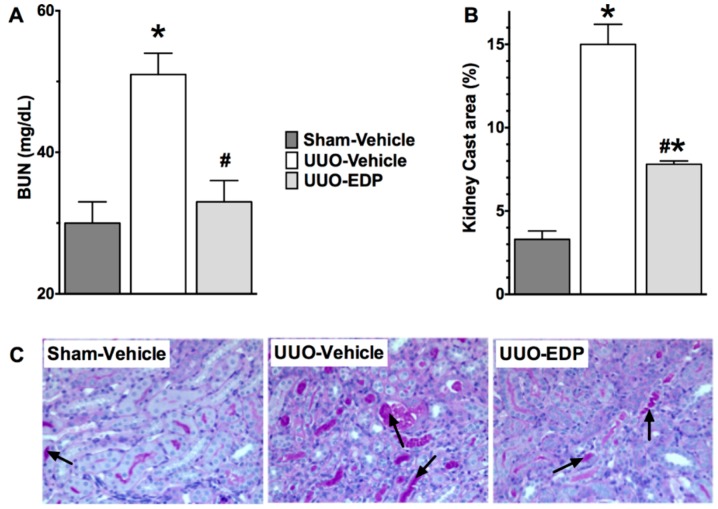
19,20-EDP treatment improved kidney function and reduced renal tubular injury in UUO by reducing blood urea nitrogen (**A**) and, tubular cast formation (**B**); representative photomicrographs (200×) showing tubular injury (arrows) in different experimental groups (**C**). All data are expressed as Mean ± SEM, * *p* < 0.05 *vs.* Sham-Vehicle, ^#^ *p* < 0.05 *vs.* UUO-Vehicle, *n* = 6–10.

UUO mice developed renal dysfunction with elevated BUN (51 ± 3 mg/dL) compared to sham control mice (30 ± 3 mg/dL). The CYP450 epoxygenase metabolite of docosahexaenoic acid, 19,20-EDP markedly reduced the blood urea nitrogen (BUN) level in UUO mice (33 ± 3 mg/dL) and reduced it to a level similar to sham control mice (Figure 1A). Serum creatinine level was elevated in vehicle treated UUO (1.3 ± 0.1 mg/dL) compared to sham-control (0.6 ± 0.09 mg/dL) mice. EDP treatment reduced serum creatinine in UUO mice (0.9 ± 0.09 mg/dL). UUO mice also developed tubular injury with marked tubular atrophy and 5-times higher amount of tubular cast formation compared to sham control. Treatment of 19,20-EDP reduced renal tubular injury in the UUO mice and also reduced tubular cast formation by 50% (Figure 1B,C).

### 2.2. Renal Fibrosis Was Reduced in 19,20-EDP Treated UUO Mice

**Figure 2:**
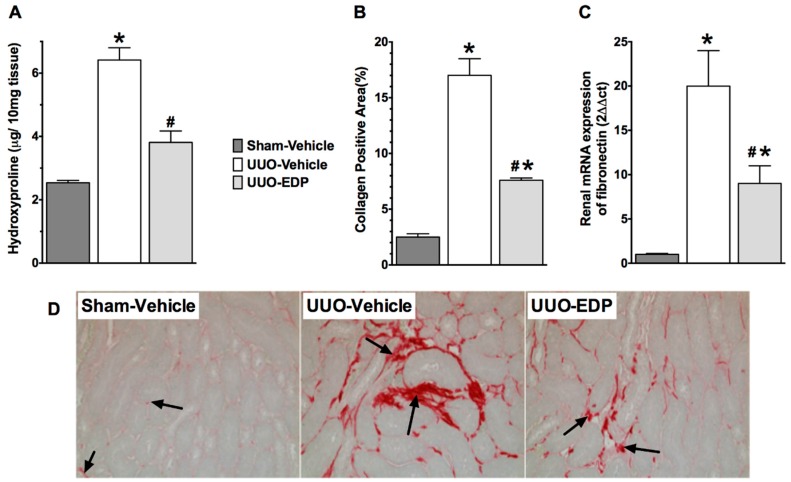
19,20-EDP treatment decreased kidney fibrosis in UUO by reducing kidney hydroxyproline content (**A**); collagen positive area in the kidney (**B**); and also by reducing kidney mRNA expression of fibronectin (**C**); representative photomicrographs (200×) showing renal interstitial fibrosis as collagen deposition (arrows) in different experimental groups (**D**). All data are expressed as Mean ± SEM, * *p* < 0.05 *vs.* Sham-Vehicle, ^#^ *p* < 0.05 *vs.* UUO-Vehicle, *n* = 6–10.

In UUO mice, there was marked renal interstitial fibrosis compared to sham control mice. Histopathological analysis of collagen positive renal fibrotic area demonstrated that UUO mice had 8-times higher collagen positive renal fibrotic area compared to sham control mice (Figure 2A,D). UUO mice treated with 19,20-EDP had reduced renal fibrosis with a 60% decrease in the collagen positive fibrotic area. (Figure 2A,D). Measuring renal tissue hydroxyproline content and fibronectin expression were also determined to evaluate renal fibrosis in UUO mice. UUO mice kidneys had elevated hydroxyproline levels (6.4 ± 0.30 μg/10 mg protein) compared to sham control mice (2.5 ± 0.05 μg/10 mg protein). 19,20-EDP treatment in UUO mice reduced the kidney hydroxyproline levels by 40% (3.8 ± 0.2 μg/10 mg protein) (Figure 2B). UUO mice also had a 20-fold higher renal mRNA fibronectin expression compared to sham control mice and 19,20-EDP reduced fibronectin expression by 50% (Figure 2C).

### 2.3. Renal Epithelial-to-Mesenchymal Transition in UUO Mice Was Reduced by 19,20-EDP Treatment

**Figure 3:**
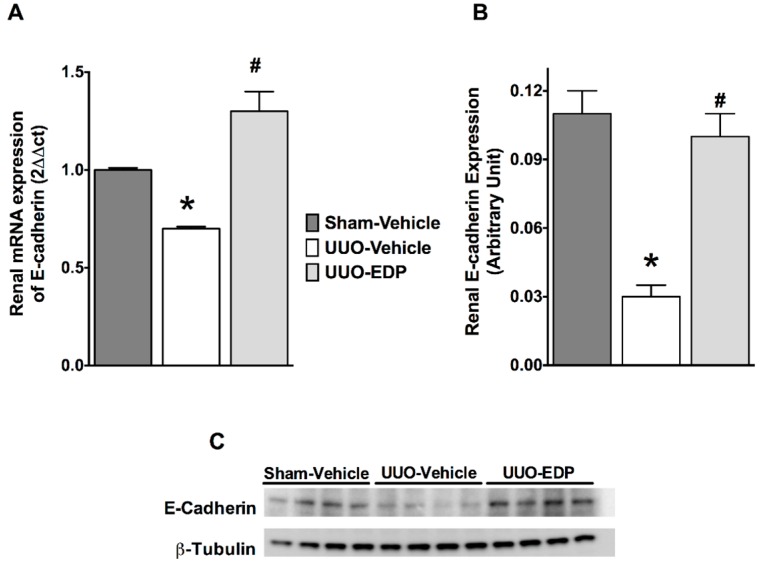
The epithelial marker E-cadherin mRNA (**A**) and protein (**B**) expressions are preserved in the kidney of UUO mice treated with 19,20-EDP; In immunoblotting experiments, the E-cadherin expression is normalized to the expression of β-tubulin. Representative immunoblots showing kidney expression of E-cadherin in different experimental groups (**C**). All data are expressed as Mean ± SEM, * *p* < 0.05 *vs.* Sham-Vehicle, ^#^ *p* < 0.05 *vs.* UUO-Vehicle, *n* = 6.

**Figure 4:**
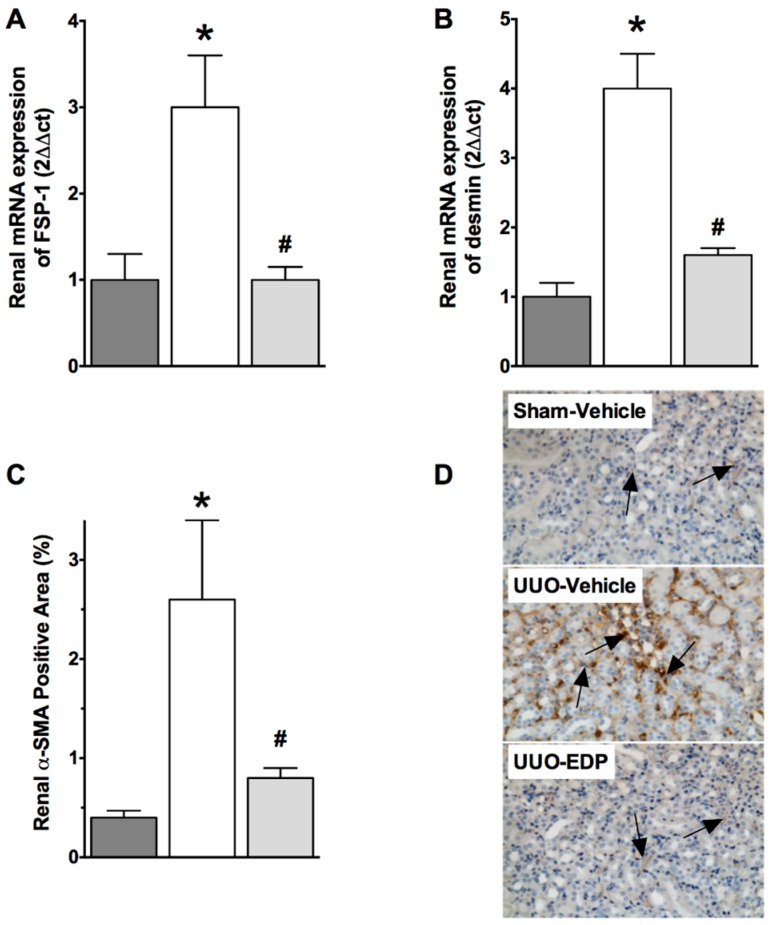
In the kidney of UUO mice mRNA expression mesenchymal marker fibroblast specific protein -1 (FSP-1) (**A**) and desmin (**B**) are reduced by 19,20-EDP treatment. 19,20-EDP treatment also reduced kidney expression of the mesenchymal marker α-smooth muscle action (α-SMA) (**C**); representative photomicrographs (200×) showing α-SMA-immunopositive areas (black arrows) in the kidney of different experimental groups (**D**). All data are expressed as Mean ± SEM, * *p* < 0.05 *vs.* Sham-Vehicle, ^#^ *p* < 0.05 *vs.* UUO-Vehicle, *n* = 6.

**Figure 5:**
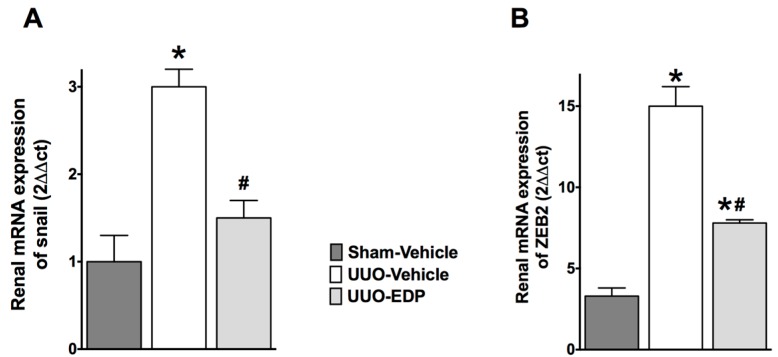
19,20-EDP treatment reduced kidney expressions of epithelial-to-mesenchymal transition regulator genes *Snail 1* (**A**) and *ZEB 2* (**B**) in UUO. All data are expressed as Mean ± SEM, * *p* < 0.05 *vs.* Sham-Vehicle, ^#^ *p* < 0.05 *vs.* UUO-Vehicle, *n* = 6.

UUO mice demonstrated epithelial-to-mesenchymal transition (EMT) compared to sham control mice. UUO mice had 30%–50% lower renal E-cadherin mRNA and protein expressions compared to sham control mice. Interestingly, in UUO mice, 19,20-EDP treatment increased E-cadherin expression by 30%–60% compared to vehicle treated UUO mice both in the mRNA and protein expression levels (Figure 3). This study also demonstrated 3–4-fold higher mRNA expression of mesenchymal markers FSP-1 and desmin in UUO mice kidneys compared to sham control mice. 19,20-EDP treatment reduced renal FSP-1 and desmin mRNA expression in UUO mice and brought their expression to a level similar to that of sham control mice (Figure 4A,B). Renal expression of a prominent EMT marker α-SMA was also markedly reduced by 19,20-EDP treatment to UUO mice. In immunohistological analysis, UUO mice demonstrated 80% higher renal α-SMA immunopositive area compared to sham control mice. 19,20-EDP treatment reduced renal α-SMA expression by 60% in UUO mice (Figure 4C,D). UUO mice also had higher renal mRNA expression of EMT regulator genes Snail 1 (3-fold higher) and ZEB2 (12-fold higher) compared to sham control mice. The 19,20-EDP treatment markedly reduced the renal expressions of *Snail 1* and *ZEB2* in UUO mice by 50%–60% (Figure 5A,B).

## 3. Discussion

Chronic kidney disease (CKD) is characterized by tubular atrophy, deposition of extracellular matrix proteins in the renal interstitium, and marked renal interstitial fibrosis, and is considered an irreversible process leading to end-stage renal diseases (ESRD) [15]. Among these pathophysiological features of CKD, renal fibrosis is considered as a hallmark of all forms of CKD that leads to renal dysfunction and to the ultimate consequence as ESRD [16]. ESRD displays as glomerular and vascular sclerosis withinterstitial expansion and collagen accumulation resulting in marked tubulointerstitial fibrosis [17]. This extracellular matrix accumulation in the tubulointerstitium ultimately leads to tubular atrophy and other changes in the kidney that contribute to a decline in renal function [18]. Kidney disease progression can be slowed by controlling diet and exercise along with anti-hypertensive drugs; however, there are currently no truly effective therapies for CKD [19,20].

In the present study, we investigated ability of 19,20-EDP, a novel CYP450 metabolite of the omega-3 fatty acid DHA to treat kidney fibrosis. We examined the anti-fibrotic action of 19,20-EDP in UUO mice that is a well-established and widely used model in investigating the mechanisms and therapeutic strategies for renal fibrosis [21]. The 19,20-EDP treatment protected the kidney from UUO-induced tubular injury and preserved renal function with a near normal BUN level. This interesting finding indicates a novel biological action for this CYP450 DHA metabolite. Our finding is in line with several earlier studies demonstrating beneficial omega-3 fatty acid kidney protective effects in a number of preclinical kidney diseases models. Omega-3 fatty acids demonstrated kidney protection in cyclosporine nephrotoxicity [22], IgA nephropathy [23] and in diabetic kidney diseases [24]. Apart from preclinical studies, epidemiological studies also suggest that omega-3 fatty acids slow the decline in renal function, e.g., prevent the decline in glomerular filtration rate in healthy elderly people [25], decrease albuminuria in type 1 diabetes [26], and reducealbuminuria in type 2 diabetes [27]. Furthermore, dietary omega-3 fatty acids have been shown to provide kidney protective effects in experimental diabetes models [28]. Our findings in the present study and these earlier findings provide plausible evidence that omega-3 fatty acids and its CYP450 metabolite, 19,20-EDP, have potential as a renal therapeutic approach.

In UUO mice, we demonstrate that along with its ability to preserve kidney function and preventing tubular injury, 19,20-EDP reduces renal fibrosis. In regards to the kidney protective action of this novel omega-3 fatty acid metabolite, this is an important finding as interstitial fibrosis is a major pathway for renal disease progression that leads end-stage renal failure. Indeed, renal interstitial fibrosis is strong histological predictor for clinical outcomes and progression to CKD [16]. We demonstrated marked reductions of collagen positive area and hydroxyproline levels in the kidney of UUO mice treated with 19,20-EDP. Similar anti-fibrotic actions have been reported for omega-3 fatty acids in several experimental renal diseases models. In a rat 5/6 nephrectomy CKD model, omega-3 fatty acid treatment markedly reduced or reversed the up-regulation of pro-fibrotic pathways and attenuated renal tubulointerstitial fibrosis [29]. Additionally, in cyclosporine nephropathy, omega-3 fatty acids demonstrated marked reduction in focal tubular atrophy and interstitial fibrosis [30]. Along with reduction of renal interstitial fibrosis these studies demonstrated that the anti-fibrotic action of omega-3 fatty acids is associated with reduced renal α-SMA expression [29,30].

The effects of omega-3 fatty acids on α-SMA expression has important implications in regards to the mechanism of renal fibrosis as α-SMA is considered a marker of mature fibroblast or myofibroblast. Indeed, myofibroblast production in the kidney constitutes the critical step leading to renal interstitial fibrosis [31], and the myofibroblast formation in the kidney is mediated by epithelial-to-mesenchymal transition (EMT). Renal EMT is considered as an important pathway and the key mechanism in the pathogenesis and progression of renal interstitial fibrosis [32]. However, apart from EMT, renal resident fibroblast and pericytes also contribute in myofibroblast formation in the kidney [33]. In EMT, loss of epithelial cell adhesion molecules, like epithelial (E)-cadherin, are switched to the mesenchymal marker α-SMA [3,34]. Indeed, studies indicate that many fibroblasts in a fibrotic kidney can originate from EMT of the tubular epithelium [35]. Overall, these earlier studies indicated that renal tubular EMT is an important event in the pathogenesis of tubulointerstitial fibrosis and inhibiting myofibroblast accumulation is critical in preventing tubulointerstitial fibrosis and preserving renal function [3,32,34].

In regards to a critical role for EMT in renal fibrosis, in the present study we demonstrate a marked down-regulation of the epithelial marker E-cadherin and an up-regulation of mesenchymal markers in the UUO kidney. Marked up-regulation of mesenchymal markers such as α-SMA and fibroblast specific protein-1 in the kidney strongly suggest a role for EMT in UUO mice. We also demonstrate that the anti-fibrotic action of 19,20-EDP in UUO mice is related to its ability to inhibit renal EMT, up-regulate epithelial marker E-cadherin, and down-regulate the mesenchymal markers α-SMA and FSP-1. Further evidence on the role for 19,20-EDP in inhibiting EMT and the associated renal fibrosis in UUO mice come from the finding that 19,20-EDP down-regulates EMT regulator genes *Snail1* and *ZEB 2*. The Snail 1 family of zinc-finger transcription factors are strong repressors of the transcription of epithelial markers and are widely implicated in both physiological and pathological EMT [36,37]. Expression of *Snail1* suppresses the epithelial marker E-cadherin transcription and enhances vimentin and fibronectin expression, leading to a full EMT phenotype, whereas silencing of *Snail1* expression reverses this process [38]. In the present study we demonstrate that renal mRNA expression of Snail 1 is up-regulated in UUO mice and 19,20-EDP treatment markedly reduced Snail 1 expression, and led us to suggest that 19,20-EDP regulates EMT in UUO mice by down regulating the transcription of Snail 1. In the present study we also demonstrate that 19,20-EDP regulates renal expression of another EMT regulator gene *ZEB2*. This transcription factor activates EMT binding to E-box elements found in the E-cadherin promoter, suppressescell-cell adhesion protein synthesis [39]. Our findings in the present study provide evidence on novel biological action of 19,20-EDP in reducing renal fibrosis by inhibiting renal EMT.

## 4. Materials and Methods

### 4.1. Chemicals

The CYP450 metabolite of epoxydocosapentaenoic, 19,20-EDP was synthesized and purified as described earlier [40]. Briefly, DHA (1 g) was epoxidized by *meta*-chloroperoxybenzoic acid (70 wt %, 380 mg, 0.5 equiv.) for 1 h. The epoxy-docosapentaenoic acid was partially purified by column chromatography eluted at 40% ethyl acetate in hexane. The 19,20-EDP was furthered purified to 100% by HPLC-based method described earlier [41]. The purity of 19,20-EDP was determined using LC/MS-MS and compared with a purified standard (Cayman Chemical Company, Ann Arbor, MI, USA) [41]. All chemicals used in this study were purchased from Sigma Aldrich (St Louis, MO, USA) unless and otherwise noted.

### 4.2. Animal Experiments

The Medical College of Wisconsin Institutional Animal Care and Use Committee approved the animal experiments conducted (Animal Protocol No. AUA 2031; Approved on 25 January 2011). Male C57Bl/6J mice (8 to 10 weeks old; Jackson Laboratories, Bar Harbor, ME, USA) were anesthetized by 2% isoflurane. After that, 6-0 silk thread was tied around the left ureter near the renal pelvis to cause complete ureter obstruction [42]. The same surgical procedure with the exception of ureter ligation was conducted in the sham mice group. Animals were housed in the Biomedical Resource Center at the Medical College of Wisconsin with a 12/12 h light–dark cycle and free access to water and rodent chow. The mice with UUO were divided into two groups. The vehicle treated UUO group (*n* = 10) received vehicle and treatment group received 19,20-EDP (1 mg/kg/day, *n* = 6). Vehicle (25% DMSO in PEG-400) and 19,20-EDP were administered continuously for the 10-day experimental period by intra-peritoneal osmotic pump (ALZET^®^osmotic pump, DurectCorporation, Cupertino, CA, USA). After 10 days of UUO or sham surgery, plasma and kidney samples were collected. The kidney samples were either fixed in 10% buffered formalin for histological studies or snap-frozen in liquid nitrogen for biochemical studies.

### 4.3. Biochemical Analysis

Blood urea nitrogen (BUN) (BioAssay Systems, Hayward, CA, USA) and serum creatinine (Cayman Chemicals, Ann Arbor, MI, USA) were measured spectrophotometrically using commercial kits. Kidney tissue hydroxyproline level was measured using a method described earlier [43]. In brief, the kidney was homogenized in 10 N HCl, hydrolyzed by autoclaving, and the lysate incubated in Chloramine T reagent (0.84% chloramines-T, 42 mM sodium acetate, 2.6 mM citric acid, and 39.5% isopropanol (pH 6.0)). The sample was then incubated in DMAB reagent (15% 4-(dimethylamino)benzaldehyde in isopropanol/perchloric acid (2:1 *vol*/*vol*)) and measured at a 550 nm wavelength.

### 4.4. Real-Time PCR Analysis

Real-Time PCR (RT-PCR) analysis was carried out to assess the renal mRNA expressions of epithelial marker epithelial E-cadherin, and a number of mesenchymal markers such α-SMA, fibroblast specific protein-1 (FSP-1), fibronectin, and desmin in the kidney tissue. Gene expression of transcription regulators Snail1 and ZEB2 were also carried out using RT-PCR analysis. According to the manufacturer’s instructions, the messenger RNA (mRNA) samples were prepared from kidney homogenate using RNeasy Mini Kit (QIAGEN, Valencia, CA, USA). The mRNA samples were quantified spectrophotometrically at a 260 nm wavelength. Total RNA (1 μg) was reverse-transcribed by iScript™ Select cDNA Synthesis Kit (Bio-Rad, Hercules, CA, USA) to cDNA. Gene expression was determined by iScript One-Step RT-PCR Kit and SYBR green using MyiQ™ Single Color Real-Time PCR Detection System (Bio-Rad Laboratories, Hercules, CA, USA). Amplified samples in wells were analyzed for homogeneity using iQ5 Optical System Software, Version 2.1 (Bio-Rad Laboratories, Hercules, CA, USA). Following denaturation at 95 °C for 2 min, 40 cycles were done at 95 °C for 10 s and at 60 °C for 30 s. Samples were run in triplicate, and the comparative threshold cycle (*C*_t_) method used to quantify fold change (2^−ΔΔ*C*t^) in target gene expression. Five to seven samples were analyzed in each experimental group.

### 4.5. Western Immunoblotting

Homogenized kidney cortex protein samples were separated by SDS-PAGE on a 10% Tris-glycine gel, and proteins were transferred electrophoretically to Polyvinylidene Fluoride (PVDF) Membrane (Bio-Rad). Blots were incubated overnight at 4 °C in a Tris NaCl buffer (TBS) containing 5% nonfat dry milk and 0.1% Tween 20 for the blocking of non-specific protein binding sites. The primary antibodies used were for E-cadherin (1:1000) (Santa Cruz Biotechnology, Santa Cruz, CA, USA). The blots were then washed in TBS-0.1% Tween, and incubated with horseradish peroxidase conjugated secondary antibody (Santa Cruz Biotechnology) for 1 h. Detection was carried out using enhanced chemiluminescence Western blot analysis and the band intensity was measured densitometrically using ImageQuant TL 8.1 image analysis software (GE Healthcare, Philadelphia, PA, USA). All densitometry values were normalized to β-tubulin (1:2000, Cell Signaling Technology, Danvers, MA, USA).

### 4.6. Histopathology

Formalin fixed renal tissue was sectioned (5 μm) and stained with Periodic Acid-Schiff (PAS) and Picrosirius Red (PSR) for histological examination. Tubular injury was determined in PAS stained tissue sections at magnification of 200× using image analyzing software by NIS Elements AR version 3.0 (Nikon instruments Inc., Melville, NY, USA). Histopathological changes were scored as published earlier [44]. PSR stained tissue sections were used to study renal interstitial fibrosis by measuring the areas positive for collagen using software by NIS Elements AR version 3.0. The collagen positive renal section areas were expressed as the percentage area fraction relative to the total area analyzed. To minimize observer bias, the tubular injury assessment (cast area calculation) and interstitial fibrosis (collagen positive area calculation) were performed by two observers in a masked fashion without knowledge of the treatment groups.

### 4.7. Immunohistopathological Analysis

Kidneys were formalin-fixed and paraffin-embedded slices deparaffinized, re-hydrated, and immunostained with anti-α smooth muscle actin (SMA) antibody (1:100; Santa Cruz Biotechnology, Dallas, TX, USA). Biotinylated rat anti-mouse secondary antibody (1:200) was used with avidin-biotinylated HRP complex (Vectastain ABC Elite kit, Vector Laboratories, Burlingame, CA, USA) and hemotoxylin used as a counterstain. Slides were then mounted and visualized by light microscopy at 400× magnification. Digital images were obtained for analysis using Nikon NIS Elements Software (Nikon Instruments Inc., Melville, NY, USA). Kidney section area positive for α-SMA was calculated by two experienced blinded reviewers using image analysis software by NIS Elements AR version 3.0 (Nikon instruments Inc.). In different experimental groups, the α-SMA positive renal section areas were expressed as the percentage area fraction relative to the total area analyzed.

### 4.8. Statistical Analysis

All data expressed as mean ± S.E.M. Two-tailed unpaired Student’s *t* test was used to determine the statistical significance between two measurements. Among groups statistical significance was determined by repeated measure one-way ANOVA followed by Tukey’s *post hoc* test using GraphPad Prism^®^ Version 4.0 software (GraphPad Software Inc., La Jolla, CA, USA). *p* < 0.05 were considered significant where the critical value of *p* was two-sided.

## 5. Conclusions

In summary, in the present study, we investigated kidney protective and anti-fibrotic effects of a novel CYP450 epoxygenase omega-3 fatty acid (DHA) metabolite in mouse model of renal interstitial fibrosis. The DHA omega-3 fatty acid metabolite 19,20-EDP demonstrates marked kidney protection and anti-fibrotic effects in UUO mice. We demonstrated that the anti-fibrotic effects of 19,20-EDP were the result of its ability to inhibit EMT in the kidney.
